# Diagnostic yield of endoscopic ultrasound-guided fine-needle aspiration-based cytology for distinguishing malignant and benign pancreatic cystic lesions: A systematic review and meta-analysis

**DOI:** 10.1371/journal.pone.0314825

**Published:** 2025-02-20

**Authors:** Cong Ding, Jian-feng Yang, Xia Wang, Yi-feng Zhou, Ye Gu, Qiang Liu, Hong-zhang Shen, Xiao-feng Zhang

**Affiliations:** 1 Department of Gastroenterology, Affiliated Hangzhou First People’s Hospital, School of Medicine, Westlake University, Hangzhou, Zhejiang, China; 2 Key Laboratory of Integrated Traditional Chinese and Western Medicine for Biliary and Pancreatic Diseases of Zhejiang Province, Hangzhou, Zhejiang, China; 3 Hangzhou Institute of Digestive Diseases, Hangzhou, Zhejiang, China; 4 Zhejiang Provincial Key Laboratory of Clinical Cancer Pharmacology and Toxicology Research, Hangzhou, Zhejiang Province, China; Hangzhou Red Cross Hospital, CHINA

## Abstract

**Background:**

Preoperative diagnosis of malignancy in patients with pancreatic cystic lesions (PCLs) remains challenging. The aim of this study was to assess the sensitivity, specificity, and positive and negative likelihood ratios (LRs) of endoscopic ultrasound-guided fine needle aspiration (EUS-FNA)-based cytology in differentiating malignant PCLs from benign PCLs.

**Methods:**

A comprehensive search was performed in multiple databases in November 2023. Studies differentiating benign and malignant PCLs via EUS-FNA-based cytology, in which the results were compared with those of surgical excision histopathology, were included in this meta-analysis. Data from the selected studies were pooled to summarize the sensitivity, specificity, positive and negative LRs, diagnostic odds ratios and summary receiver operating characteristic (SROC) curves.

**Results:**

We included 755 patients from 15 distinct studies who underwent EUS-FNA-based cytology and had a histopathological diagnosis. The pooled sensitivity and specificity in diagnosing malignant PCLs were 0.62 (95% CI, 0.42–0.78) and 0.96 (95% CI, 0.91–0.98), respectively. The positive and negative LRs for diagnosing malignant PCLs were 16.3 (95% CI, 7.2–37.0) and 0.40 (95% CI, 0.25–0.64), respectively. The area under the curve (AUC) was 0.94 (95% CI, 0.91–0.95).

**Conclusions:**

EUS-FNA-based cytology has overall high specificity, medium sensitivity and good diagnostic accuracy in differentiating malignant from benign PCLs. Further research is needed to improve the overall sensitivity of EUS-FNA-based cytology for the diagnosis of malignant PCLs.

## Introduction

With the development of imaging techniques, pancreatic cystic lesions have become more commonly detected. Some PCLs comprise a heterogeneous group of diseases with varying degrees of malignancy, but some PCLs are completely benign. Clarifying the nature of cystic lesions can not only help with avoiding unnecessary surgery for benign lesions and preventing overtreatment and postoperative complications but can also improve prognosis by facilitating earlier malignant lesion detection and surgical treatment. However, there are still many challenges in diagnosing PCLs as benign or malignant before surgery. EUS-FNA is a minimally invasive diagnostic method that can provide investigators with cyst fluid for chemical and cytological analyses. The international consensus guidelines [1] for the management of PCLs have proposed including examinations via EUS-FNA. Numerous retrospective and prospective case series on EUS-FNA of cystic pancreatic lesions have been published. Therefore, the aim of this study was to perform a structured meta-analysis of the available evidence on the diagnostic accuracy of EUS-FNA-based cytology in differentiating malignant PCLs from benign PCLs.

## Materials and methods

This systematic review and meta-analysis was conducted in adherence to the Preferred Reporting Items for Systematic Reviews and Meta-Analyses (PRISMA) Statement and was registered with the PROSPERO International Prospective Register of Systematic Reviews (registration number: **CRD42023388959**).

### Literature search

The literature search strategy was based on the following inclusion criteria:

1. analyzed cytology from EUS-FNA for the diagnosis of pancreatic cystic lesions;
2. participants >18 years of age;
3. sample size >10;
4. surgical histology as the gold standard;
5. published in English; and
6. presented sufficient data for the calculation of sensitivity and specificity.

We excluded studies that met any of the following criteria:

1. review articles, case reports, letters to the editor, or animal models; or
2. studies that did not analyze cytology results but instead were based on conclusions solely derived from EUS-FNA cyst fluid analysis.

The literature search was conducted on November 8, 2023, using medical databases such as PubMed, Embase, and Cochrane. The full search strategy for PubMed is available in Table 1. In addition, we screened the reference lists of eligible studies and relevant reviews to identify any additional studies that met our inclusion criteria. The search results were managed via the citation software Endnote.

**Table 1:** Search strategy on PubMed electronic database.

Search	Query	Items found
**#7**	#3 and #6	407
**#6**	#4 OR #5	5916
**#5**	(Endoscopic Ultrasound Guided Fine Needle Aspiration[Title/Abstract]) OR (EUS-FNA[Title/Abstract])	3572
**#4**	"Endoscopic Ultrasound-Guided Fine Needle Aspiration"[Mesh]	3586
**#3**	#1 OR #2	8588
**#2**	((Cyst, Pancreatic[Title/Abstract]) OR (Cysts, Pancreatic[Title/Abstract])) OR (Pancreatic Cysts[Title/Abstract])	1574
**#1**	"Pancreatic Cyst"[Mesh]	8046

### Study selection process and data extraction

Two independent investigators reviewed the study titles and abstracts, and studies that satisfied the inclusion criteria were retrieved for full-text assessment. The reasons for the exclusion of studies were documented. Any disagreements between the two researchers were resolved by consensus or by consultation with a third researcher. If there were multiple publications of the same study, we included the most recent and comprehensive report with the largest sample size. One reviewer extracted the following data from the selected studies: first author name, year of publication, total number of patients, number of subjects who underwent analysis, research center, study design, study duration, needle type, cyst type, and basic methods for final diagnosis. A second reviewer checked all the information for accuracy, and if there was any disagreement, a consensus was reached. If relevant information was missing from the articles, we contacted the corresponding authors to obtain the data. We excluded studies from the meta-analyses if we were unable to obtain relevant data. All contacts with the authors were documented.

### Definitions and outcomes

The gold standard for classification is resected cyst histology. Malignant cysts include mucinous cystic neoplasms (MCNs) and intraductal papillary mucinous neoplasms (IPMNs) with high-grade dysplasia (HGD) or invasive carcinoma (INV), cystic pancreatic neuroendocrine tumors, and solid pseudopapillary neoplasms. Benign cysts included pseudocysts, serous cystadenomas, lymphoepithelial cysts, duplication cysts, and MCNs and IPMNs with low-grade dysplasia or intermediate-grade dysplasia. The cytology reports were categorized as follows: 1) malignant or suspicious for malignancy (positive for malignancy); 2) benign or atypical (negative for malignancy); and 3) nondiagnostic (excluded from the analysis). Two independent reviewers assessed the risk for bias according to the PRISMA recommendations.

### Statistical analysis

All the statistical analyses were conducted via STATA15.0 and RevMan version 5 from the Cochrane collaboration, and the results are expressed as rates and 95% confidence intervals (CIs). The assessment of the risk of bias and quality of the included studies was conducted by one researcher supervised by the second researcher via the modified QUADAS-2 tool. This tool consists of four domains: patient selection, index test, reference standard, and flow and timing. Each domain was assessed in terms of the risk of bias. The first three domains were also assessed in terms of applicability. Three judgment criteria (‘Low risk’; ‘High risk’; and ‘Unclear risk’) were recorded for each domain of the included studies. Disagreements were resolved through consultation with a third reviewer.

Sensitivity was computed as the proportion of positives correctly identified with the test (TPs) on the prevalence of disease in the study cohort [(TPs + false negatives (FNs)]. Specificity was calculated as the proportion of negatives correctly identified as such (TNs) among the patients who were not affected by the disease in the study cohort [(TNs + false positives (FPs)]. The likelihood ratio for a positive test result (LR+) was calculated by dividing the sensitivity by the false-positive error rate, whereas the likelihood ratio for a negative test result (LR−) was calculated by dividing the false-negative error rate by the specificity. The diagnostic odds ratio (DOR) was calculated by dividing (LR+) by (LR−). The diagnostic score was calculated as the true positive rate minus the false positive rate.

Cochran’s Q and Higgins I-squared statistic were calculated to estimate the heterogeneity of the included studies. I^2^ = 0% was considered to indicate no observed heterogeneity, and I^2^>50% was considered to indicate substantial heterogeneity. A summary receiver operating characteristic (SROC) curve was drawn, and the area under the curve (AUC) was reported. The following guidelines have been suggested for the interpretation of intermediate area under the ROC curve (AUROC) values: low (0.5≥AUC≤0.7), moderate (0.7≥AUC≤0.9), or high (0.9≥AUC≤1) accuracy. Meta-regression analysis was applied to explore the origin of heterogeneity. Any potential publication bias was verified through visual assessment of funnel plots. For all the statistical methods used in the meta-analysis, a P value<0.05 was considered to indicate statistical significance.

## Results

### Systematic review

**Fig 1:**
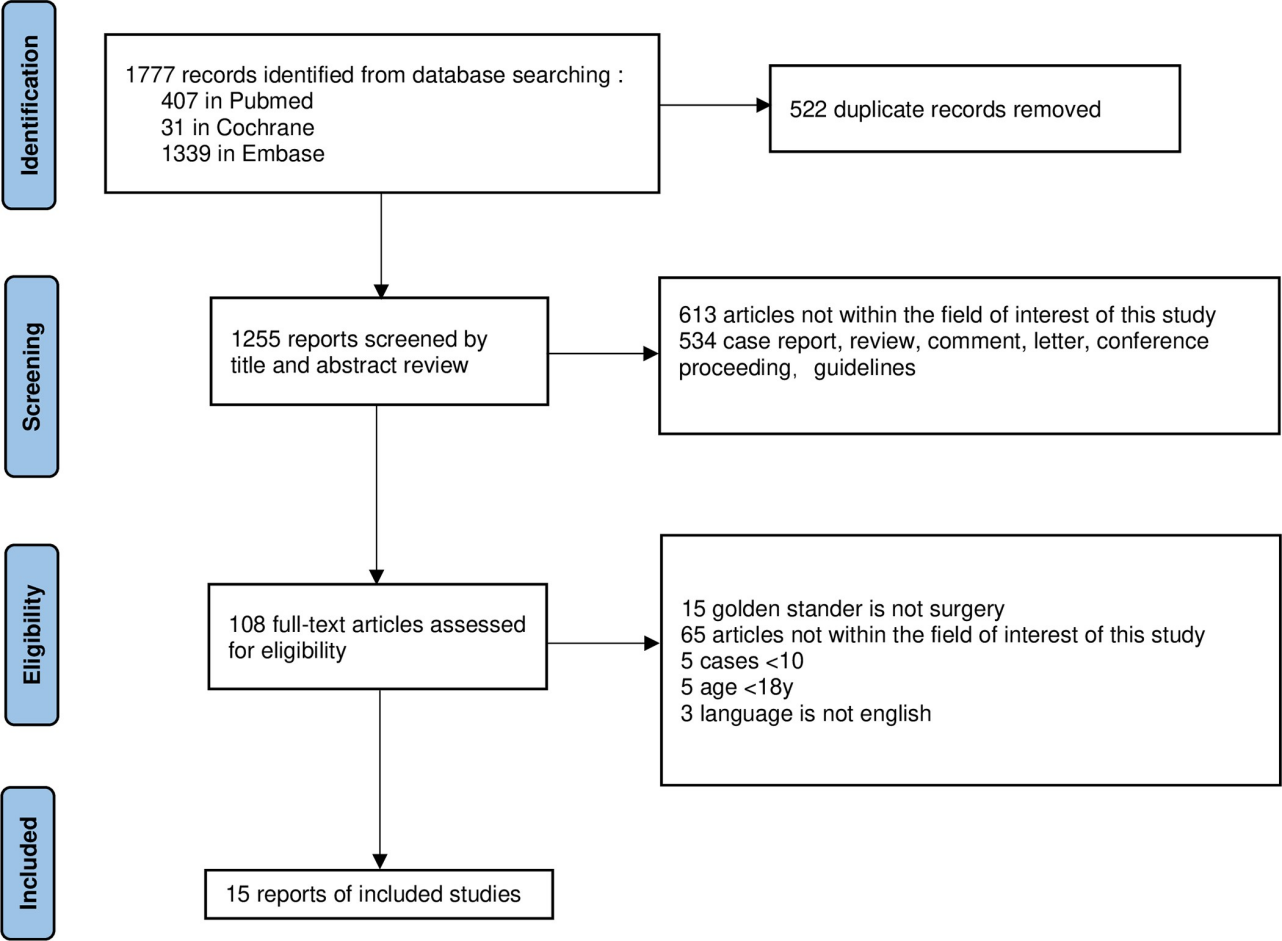
Flow diagram of the study selection process for the meta-analysis.

Our study selection process is described in detail in Fig 1. The initial literature search generated 1777 results. After removing duplicate records (522 studies) and screening titles and abstracts (1255 records), we assessed 108 full-text studies, leaving 15 articles [2–16] included in our current meta-analysis.

Two studies (Chen2021 [2] and Chen2017 [5]) were from the same research center, with partial overlap in study time; Chen2021 included a total of 40 cases of 19G puncture needles, and study Chen2017 included a total of 75 cases of 19G and 22G puncture needles; tp, fp, fn, and tn in study Chen2021 were 27, 2, 1, and 10 and corresponded to 1, 1, 1, and 72 in study Chen2017, respectively. Because there were at least 27 cases (27/40) in the Chen2017 study that were not included in the Chen2021 study and at least 62 cases (62/75) in the Chen2021 study that were not included in the Chen2017 study; both studies were included in the analysis.

The 15 studies included in the present analysis were published between 2002 and 2021 and included a total of 755 patients (Table 2). Six of the 15 papers were prospective [4, 5, 10, 14–16], and the remaining 9 were retrospective [2, 3, 6–9, 11–13]. All the studies were single-center studies. Eleven studies included almost all types of PCLs [2–7, 9, 10, 12, 15, 16], 3 studies included only mucinous cysts [8, 11, 13], and 1 study excluded IPMNs [14]. Seven studies spanned a period longer than 5 years [4, 6, 7, 9, 12–14], and 6 studies were shorter than 5 years [2, 3, 5, 8, 15, 16]. 19G puncture needles were not used in 6 studies [3, 12–16] but were used in 5 studies [2, 5, 6, 8, 10]. The characteristics of the studies included in the present meta-analysis are presented in Table 2.

**Table 2:** Characteristics of included studies.

Study	Title of the publication	Country	centers	design	Needle	cyst type	duration(months)	No. patients	No. patients under analysis	No. malignancy in diagnostic patients	No. malignancy in nondiagnostic patients	TP	FP	FN	TN
Chen2021	Surgical Endoscopy	China	single center	retrospective	19G	PCLs	60	40	40	29	0	27	2	1	10
Ali2019	Oncotarget	USA	single center	prospective	NA	PCLs	84	46	36	18	0	11	7	2	16
Chen2017	World Journal of Gastroenterology	China	single center	prospective	19G,22G	PCLs	18	75	75	2	0	1	1	1	72
Megan2015	Journal of the Pancreas	USA	single center	retrospective	19G,22G,25G	PCLs	72	40	27	3	2	2	1	6	18
Ivan2014	Cancer Cytopathology	USA	single center	retrospective	NA	PCLs	72	35	26	9	3	7	2	1	16
Judy2012	Journal of Gastrointestinal Surgery	USA	single center	retrospective	NA	PCLs	169	120	88	8	3	4	4	10	70
Xian2012	Journal of Digestive Diseases	China	single center	retrospective	19G,22G	MC	53	20	20	6	0	6	0	4	10
Koller2021	Gastroenterologie a Hepatologie	Slovakia	single center	retrospective	22G,25G	PCLs	36	10	5	1	1	0	1	2	2
Koen2012	Scandinavian Journal of Gastroenterology	Netherlands	single center	prospective	19G,22G	PCLs	NA	30	13	1	0	1	0	3	9
Martha2010	Cancer Cytopathology	Canada	single center	retrospective	NA	MC	NA	112	112	11	0	11	0	28	73
Ajay2008	Annals of Surgical Oncology	USA	single center	retrospective	22G	PCLs	90	29	16	4	3	4	0	3	9
Shireen2007	Clinical Gastroenterology and Hepatology	Indiana	single center	retrospective	22G	MC	112	65	58	19	2	15	4	3	36
Siriboon2007	Journal of the Pancreas	USA	single center	prospective	22G	PCLs *	108	48	34	5	0	4	1	2	27
Jean2003	American Journal of Gastroenterology	France	single center	prospective	22G	PCLs	48	67	67	15	0	15	0	1	51
Robert2002	Gastrointestinal endoscopy	USA	single center	prospective	22G	PCLs	38	18	15	3	2	3	0	6	6

NA, not available; PCLs, pancreatic cystic lesions; MC, mucinous cystic lesions; TP, true positive; FP, false positive; FN, false negative; TN, true negative

*, PCLs excluded IPMN

### Quality of studies

**Fig 2:**
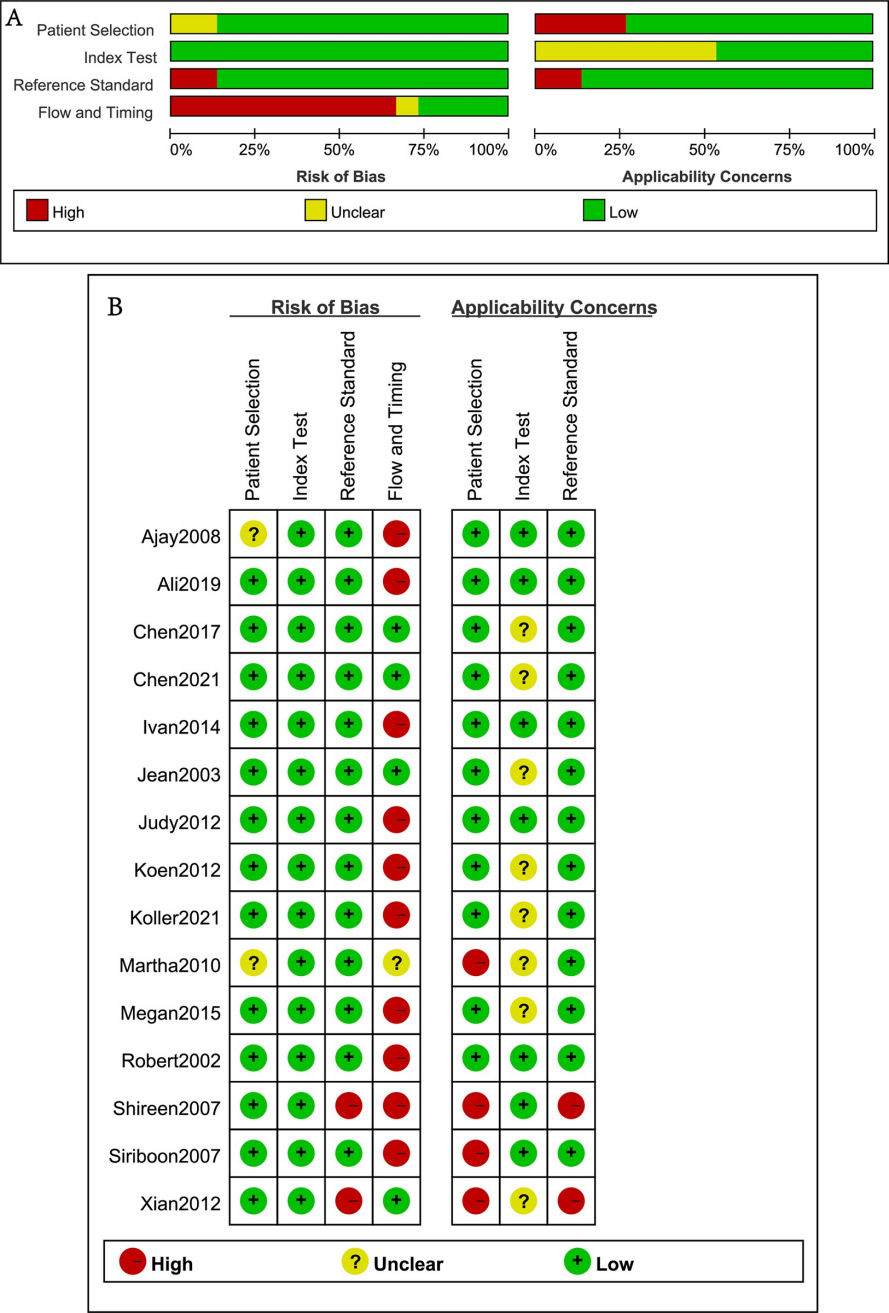
Quality assessment: Quality assessment of the studies by QUADAS-2. (A) Methodological quality graph. (B) Methodological quality summary.

QUADAS-2 criteria were adopted for evaluating the quality of the included studies. The domains were evaluated by risk of bias and concerns regarding applicability and are graphically displayed in Fig 2.

In most of the studies, the risk of patient selection bias was low. Two studies did not provide the duration of the trial, resulting in an unknown continuity of cases [11, 12]. Most of the studies showed a high risk of bias regarding flow and timing because some patients were excluded from the meta-analysis, as cytology diagnosis was classified as no diagnosis [3, 4, 6, 7, 9, 10, 12–14, 16]. The risk of index test bias was low in all of the studies. The risk of reference standard bias was high in 2 studies that defined malignant lesions as infiltrating lesions only, and HGD was not included in malignant lesions [8, 13].

Applicability concerns regarding patient selection were reported to be high for 4 studies because they did not include all kinds of PCLs, and there were mismatches between the study setup and review questions [8, 11, 13, 14]. Applicability concerns for the index test were reported to be uncertain for 8 studies because the implementation or setting of the index test did not match the review questions [2, 3, 5, 6, 8, 10, 11, 15]. Finally, applicability concerns regarding the reference standard were reported to be high for 2 studies because HGD was not included as a malignant lesion [8, 13].

### Meta-analysis

**Fig 3:**
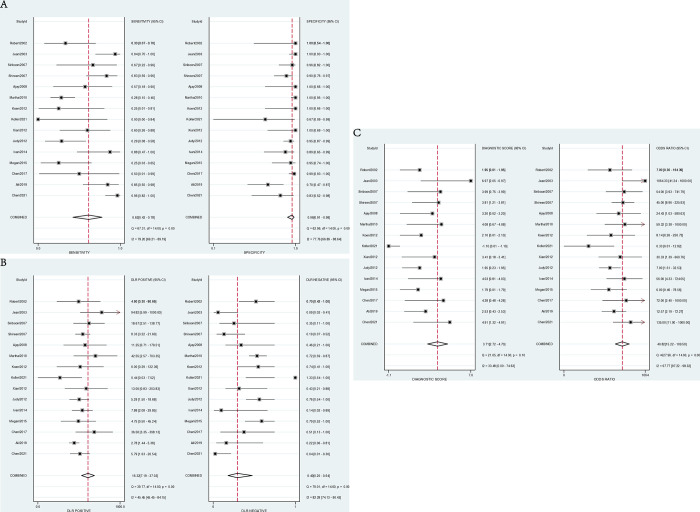
Forest plots for cytology. (A) Sensitivity and specificity. (B) Positive likelihood ratio and negative likelihood ratio. (C) Diagnostic odds ratio and diagnostic score.

**Fig 4:**
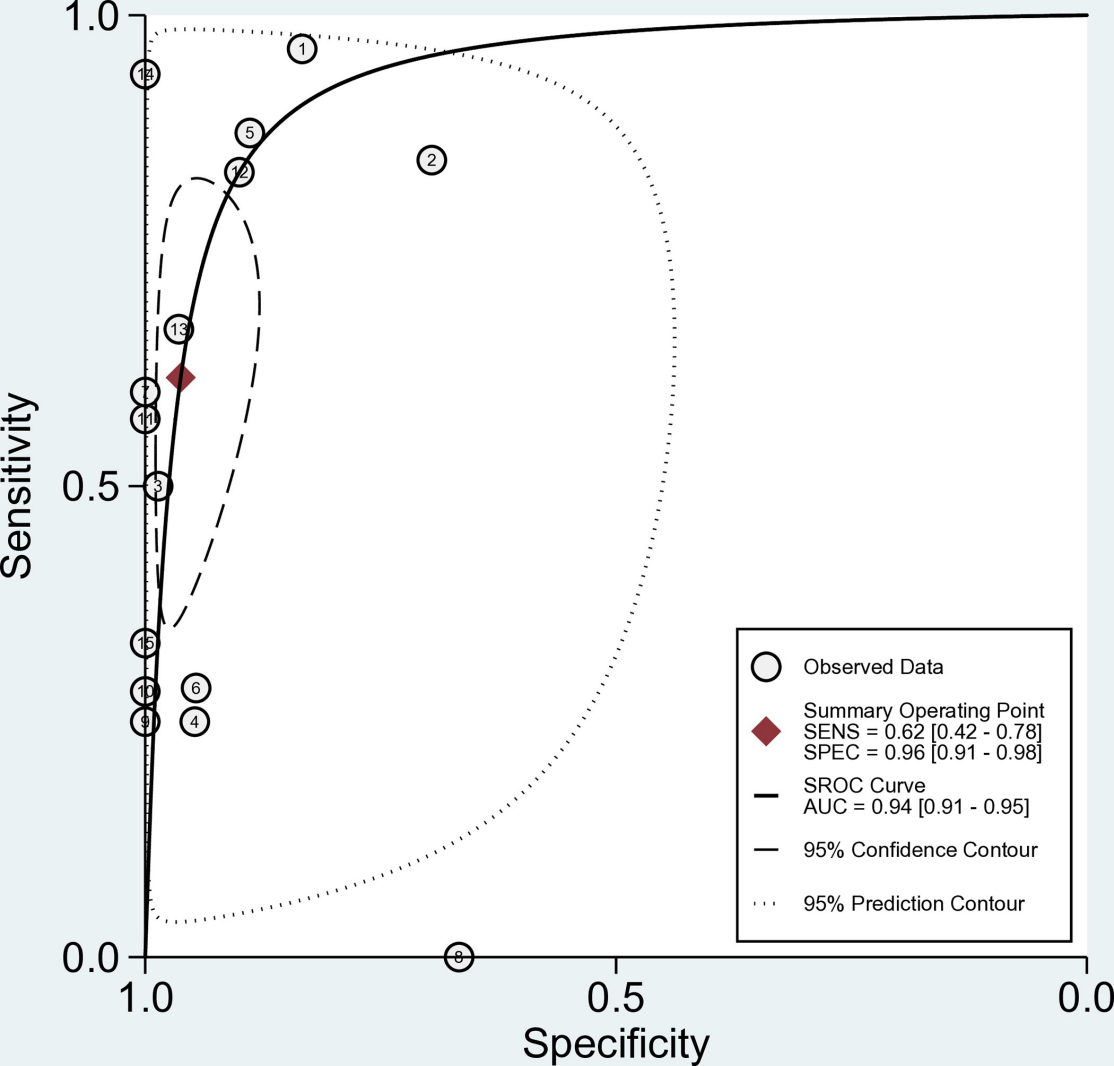
Summary receiver operating characteristic (SROC) curves for all studies included in the meta-analysis.

The forest plots of the sensitivity, specificity, PLR, NLR and DOR of EUS-FNA-based cytology for distinguishing malignant PCLs are shown in Fig 3. Point estimates were plotted with 95% CIs for each cohort. For EUS-FNA-based cytology, the pooled sensitivity and specificity were 0.62 (95% CI, 0.42–0.78) and 0.96 (95% CI, 0.91–0.98), respectively. The pooled PLR and NLR were 16.30 (95% CI, 7.20–37.00) and 0.40 (95% CI, 0.25–0.64), respectively. The DOR was 41.0 (95% CI, 15.0–110.0). The pooled rate of sample adequacy was 0.85 (95% CI, 0.73–0.94). An I^2^>50% suggested heterogeneity among the studies. An SROC curve was generated, and the AUC was 0.94 (95% CI, 0.91–0.95), which illustrated relatively high diagnostic value (Fig 4).

### Subgroup analysis and meta-regression

To explore the source of heterogeneity, a meta-regression analysis was performed (Table 3). We identified several potential sources of heterogeneity: prospective versus retrospective design, design duration longer than 5 years or not, all kinds of PCLs versus mucinous cysts, and needle type, which included 19G or excluded 19G. Meta-regression did not reveal statistically significant differences in sensitivity between subgroups. We assume that this may be because most of the included studies had a small sample size. In addition, factors such as lesion size, lesion location, and endoscopist experience may be sources of heterogeneity but could not be analyzed because of missing data. However, for specificity, the study length variable was statistically significant (P = 0.01), indicating that there was a statistically significant difference in the specificity obtained when studies with a time span longer than 5 years and those shorter than 5 years were included.

**Table 3:** Predefined subgroup analysis with 95% confidence intervals and P values.

Subgroup	No. of studies	Pooled sensitivity	p	Pooled specificity	p
Study design			0.88		0.68
Prospective	6	0.66(0.37–0.94)		0.97(0.94–1.00)	
Retrospective	9	0.60(0.37–0.83)		0.95(0.91–1.00)	
Study length					
<5 years	6	0.71(0.46–0.97)	0.62	0.98(0.95–1.00)	0.01
≥.01(0.9	7	0.65(0.40–0.89)		0.91(0.86–0.97)	
Cystic type			0.85		
All	12	0.62(0.42–0.83)		0.96(0.91–1.00)	0.23
Mucinous cyst	3	0.59(0.21–0.97)		0.98(0.94–1.00)	
Needle type			0.75		
Include 19G	5	0.59(0.25–0.93)		0.97(0.93–1.00)	0.95
Exclude 19G	6	0.66(0.37–0.94)		0.97(0.93–1.00)	

### Publication bias analysis

**Fig 5:**
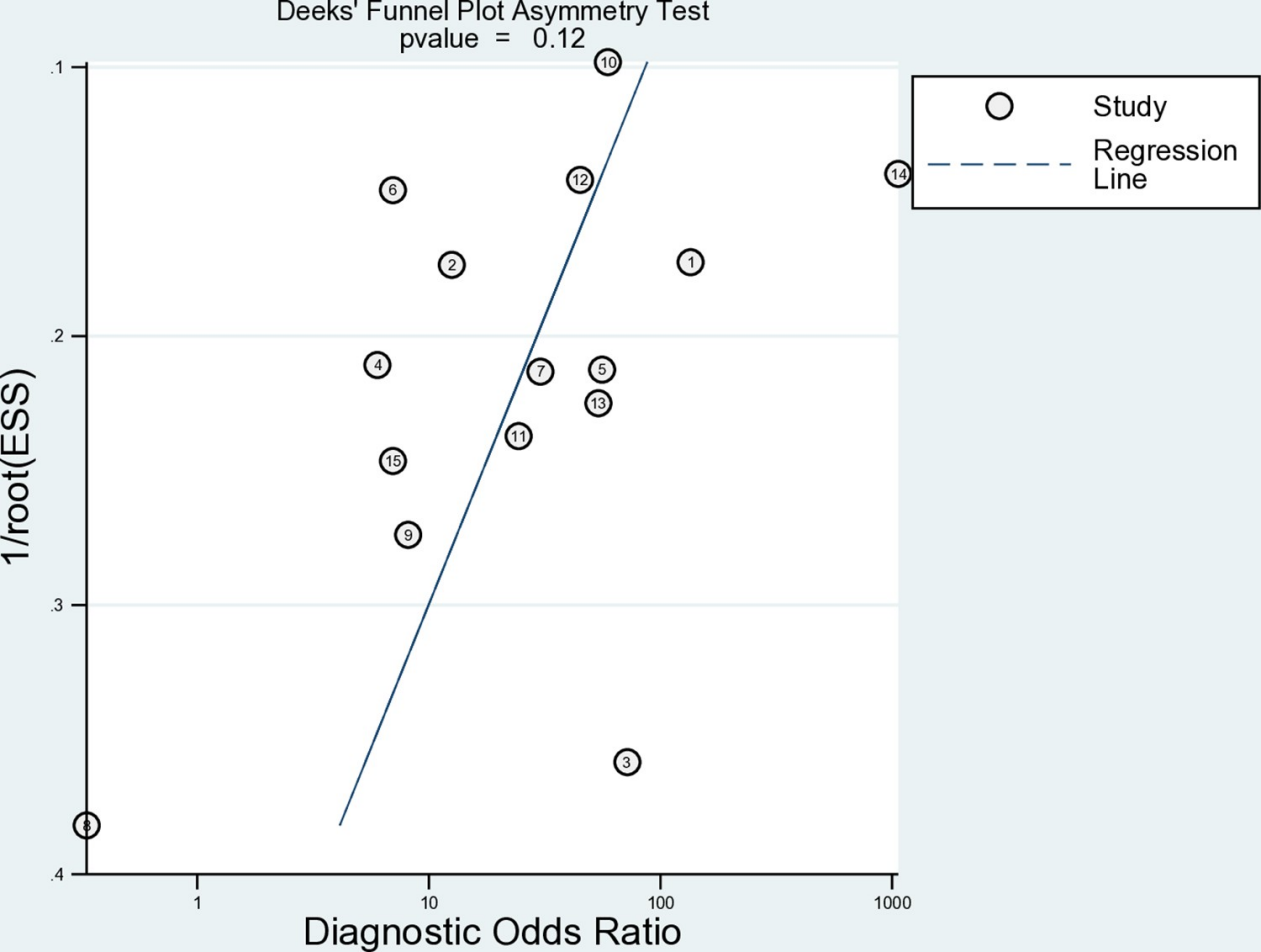
Funnel plot showing publication bias of the studies included in the meta-analysis.

The funnel plots (Fig 5) were not statistically significant (p = 0.12), indicating that publication bias did not affect the pooled diagnostic accuracy of this meta-analysis.

## Discussion

Pancreatic cystic lesions include a variety of benign and malignant lesions. Determining the nature of PCLs and selecting patients with high-risk pathologic subtypes for surgery while avoiding excessive follow-up or unnecessary surgery in patients with benign lesions remain challenging. EUS-FNA, which allows for a cytologic diagnosis, is an important minimally invasive method to clarify the nature of the lesion preoperatively. In this study, we investigated the diagnostic value of EUS-FNA cytology for the diagnosis of benign and malignant pancreatic cystic lesions.

This meta-analysis revealed an AUC of 0.94 (95% CI, 0.91–0.95) and a sensitivity and specificity of 0.62 (95% CI, 0.42–0.78) and 0.96 (95% CI, 0.91–0.98), respectively, for diagnosing malignant pancreatic cysts. The pooled positive and negative LRs for the diagnosis of malignant PCLs were 16.3 (95% CI, 7.2–37.0) and 0.40 (95% CI, 0.25–0.64), respectively, so EUS-FNA cytology had high specificity and diagnostic accuracy for the diagnosis of malignant lesions. A meta-analysis [17] that included a total of 18 studies comprising 1024 patients revealed that the pooled sensitivity was 51%, the specificity was 94%, the DOR was 23.91 (14.09–40.59), and the AUC was 0.90, indicating that EUS-FNA is valuable in diagnosing malignant PCL. However, the above study was published in 2015, and the diagnostic gold standard was partial clinical follow-up without surgical pathologic confirmation. Our meta-analysis included 5 recently published articles, all samples in our article have surgical pathology as the gold standard, and our pooled specificity in diagnosing malignant PCLs was 0.96 which was similar as the above article, while our pooled sensitivity was 0.62 which was higher than the above article.

In a study [18] by Van et al., the sensitivity of cytology in the diagnosis of malignant mucinous cystic lesions was only 22%, and in another study [19], cytology was more specific than imaging for detecting malignancies in cysts greater than 3 cm in size. Although cytologic analysis of cyst fluid provides a specific diagnosis, the sensitivity and adequacy of this technique are limited. This is due to several factors. First, insufficient simple volume and cyst fluid lack the necessary cellular contents to make a diagnosis. Second, cyst fluid aspirates often contain gastrointestinal contaminants from the needle tract.

Some measures could be taken to improve the cytologic results. EUS-FNA of the cyst wall or its solid component (mural nodule or mass lesion) led to a significant increase in diagnostic yield. Lim et al. [20] reported that in the absence of a solid component, the cytology yield was 28.8%, whereas if the mural nodule was sampled via FNA, the yield was 78%. Maria et al. [21] reported that cytological specimens from PCLs obtained via EchoBrush at the time of EUS are superior to those obtained via conventional EUS-FNA (85.1% vs. 66.3%), mainly because of the greater yield of epithelial cells. A meta-analysis [22] suggested that smear cytology (SC) with rapid onsite evaluation was a superior diagnostic technique for EUS-FNA examination of pancreatic lesions. If rapid onsite evaluation is not possible, liquid-based cytology (LBC) may be used in place of smear testing. However, a meta-analysis [23] showed that if the SC and LBC results are inconsistent, endoscopic ultrasound-guided through-the-needle biopsies (TTNBs) have greater sensitivity and specificity for pancreatic lesions than cytology does; for TTNBs, the pooled sensitivity was 0.78 (95% CI 0.61–0.89), the specificity was 0.99 (95% CI 0.90–0.99), and the AUC was 0.92 for the diagnosis of high-risk cysts.

Analysis of cyst fluid by biochemical and genetic testing could add significant value to an accurate cytologic diagnosis. Mutations such as those in PTEN, p53, and SMAD4, which are known to occur in advanced stages of malignancy, may be useful in supporting the cytologic diagnosis of high-risk cysts [24]. As a simple and feasible detection method, serum tumor markers (STMs) may be used to predict advanced cystic mucinous neoplasms (A-cMNs). Sun et al. [25] reported that CA19-9 exhibited the highest sensitivity (SE) (54.2%) and accuracy (76.5%) and a moderate ability (AUC = 0.766) to predict A-cMNs, whereas CEA, CA125, and CA724 had a low ability to predict A-cMNs. The combination of STMs improved the SE to 62.5%. Combination testing is the future trend of diagnostic PCL protocols, and samples can be from cystic fluid, punctured cell tissues, blood, feces and so on. Combined with detection via proteomics, genomics, miRNA and other biomarkers, cytology and imaging may improve the accuracy of PCL classification and malignancy diagnosis. There were several limitations to our analysis. Most of the studies were retrospective and were conducted in a single center. Some important factors, such as cyst size and location, were not provided in each study, which made further analysis difficult. Third, there were no reports of complications during the procedures in the included articles.

## Conclusions

Our meta-analysis revealed that EUS-FNA-based cytology has overall high specificity, medium sensitivity and good diagnostic accuracy in differentiating malignant from benign PCLs. EUS-FNA is therefore likely to play an increasingly important role in the investigation of cystic pancreatic lesions. Furthermore, the combination of other methods may improve the overall accuracy of EUS-FNA in the diagnosis of PCLs.

## Supporting information

S1 Checklist Completed PRISMA 2020 checklist. (DOCX)

S1 Raw data The raw data. (XLSX)

S1 File Descriptions of included and excluded records and reasons. (XLSX)
